# The R2 non-neuroinvasive HSV-1 vaccine affords protection from genital HSV-2 infections in a guinea pig model

**DOI:** 10.1038/s41541-020-00254-8

**Published:** 2020-11-06

**Authors:** David I. Bernstein, Rhonda D. Cardin, Gregory A. Smith, Gary E. Pickard, Patricia J. Sollars, David A. Dixon, Rajamouli Pasula, Fernando J. Bravo

**Affiliations:** 1 Cincinnati Children’s Hospital Medical Center, University of Cincinnati, Cincinnati, OH USA; 2 School of Veterinary Medicine, Louisiana State University, Baton Rouge, LA USA; 3 Department of Microbiology-Immunology, Northwestern University Feinberg School of Medicine, Chicago, IL USA; 4 School of Veterinary Medicine and Biomedical Sciences, University of Nebraska, Lincoln, NE USA

**Keywords:** Live attenuated vaccines, Preclinical research

## Abstract

Herpes simplex virus (HSV) infections are common and can cause severe illness but no vaccine is currently available. The recent failure of subunit HSV vaccines has highlighted the need for vaccines that present a diverse array of antigens, including the development of next-generation live-attenuated vaccines. However, most attenuated HSV strains propagate poorly, limiting their ability to elicit protective immune responses. A live-attenuated vaccine that replicates in non-neural tissue but is ablated for transmission into the nervous system may elicit protective immune responses without evoking neurologic complications or establishing life-long infections. Initial studies of R2, a live-attenuated vaccine that is engineered to be unable to invade the nervous system, used the guinea pig genital HSV model to evaluate the ability of R2 to replicate at the site of inoculation, cause disease and infect neural tissues. R2 was then evaluated as a vaccine using three routes of inoculation: intramuscular (IM), intradermal (ID) and intravaginal (IVag) and compared to IM administered gD2+MPL/Alum vaccine in the same model. R2 replicated in the genital tract but did not produce acute or recurrent disease and did not infect the neural tissue. The R2 vaccine-induced neutralizing antibody and decreased the severity of acute and recurrent HSV-2 disease as well as recurrent shedding. The ID route was the most effective. ID administered R2 was more effective than gD2+MPL/Alum at inducing neutralizing antibody, suppressing acute disease, and acute vaginal virus replication. R2 was especially more effective at reducing recurrent virus shedding, the most common source of HSV transmission. The live-attenuated prophylactic HSV vaccine, R2, was effective in the guinea pig model of genital HSV-2 especially when administered by the ID route. The use of live-attenuated HSV vaccines that robustly replicate in mucosal tissues but are ablated for neuroinvasion offers a promising approach for HSV vaccines.

## Introduction

The development of an effective vaccine for herpes simplex virus (HSV) is a priority^1–3^ because it is a common infection that causes physical and emotional stress as well as increasing the risk for HIV infection^4–6^. HSV is a neuroinvasive pathogen that rapidly transmits into the peripheral nervous system following replication in exposed mucocutaneous sites^7^. HSV can also invade the central nervous system and cause life-threatening encephalitic infections^8^, and may also contribute to dementias such as Alzheimer’s disease^9,10^. Although HSV type 1 (HSV-1) is the most prevalent form of the virus, worldwide about 400 million people are infected with HSV type 2 (HSV-2), the predominant cause of genital herpes^11^. Both HSV-1 and HSV-2 can also cause neonatal herpes, a devastating disease most often acquired during birth from infected mothers^12^. HSV-1 and HSV-2 also cause blindness as well as severe disseminated infections in immunocompromised individuals.

There are a number of approaches that have been taken to develop an HSV vaccine including sub-unit vaccines, peptide vaccines, live-attenuated vaccines, inactivated-whole-virus vaccines, DNA vaccines, disabled single-cycle vaccines, and vectored vaccines^1–3^. Most recent clinical trials have used the HSV glycoproteins, in particular HSV-2 glycoprotein D (gD2), administered with a potent adjuvant such as MPL/Alum^13,14^. Although the initial trials of the latter were promising, at least in HSV seronegative women^13^, a larger trial in seronegative women failed to protect from HSV-2 but did demonstrate some protection against HSV-1^14^.

The desire to present a more diverse set of HSV antigens has led to a renewed interest in live-attenuated HSV vaccines. The success and extended safety record of another live-attenuated alpha-herpesvirus vaccine for varicella-zoster virus (VZV) has provided further encouragement for this approach^15,16^. The R2 vaccine evaluated here is a live-attenuated HSV-1 strain encoding pUL37 tegument protein mutated in region 2, which is essential for neuroinvasion^17^. Mutation of this region renders HSV-1, and the related veterinary pathogen pseudorabies virus (PRV), incapable of spreading by retrograde axonal transport to peripheral ganglia both in culture and animals without compromising viral propagation and spread at the site of inoculation^17^. Consistent with the axonal transport deficit, the R2 attenuated viruses were specifically ablated of neuroinvasive potential and were avirulent in mice^17^. Thus, the R2 approach showed potential as a new live-attenuated HSV vaccine capable of amplified presentation of the entire cohort of HSV antigens in the absence of nervous system complications or the establishment of latent infections. Indeed, HSV-1 and PRV mutated in the R2 effector region of pUL37 were highly immunogenic and prevented subsequent challenges with wild-type viruses from invading the nervous system, establishing latency, or causing ocular or encephalitic disease in mice^17^. The PRV R2 vaccine also protected pigs from high-dose virulent challenge, demonstrating the efficacy of the R2 design in a native host^18^.

In this report, we used the guinea pig model of genital HSV-2 to test the efficacy of R2 vaccination. This model includes the assessment of acute and recurrent HSV disease and virus shedding. The ability of the HSV-1 R2 virus to invade neural tissue from the genital tract and to provide protection from subsequent intravaginal (IVag) HSV-2 challenge were assessed.

## Results

### Lack of R2 virulence

**Fig. 1:**
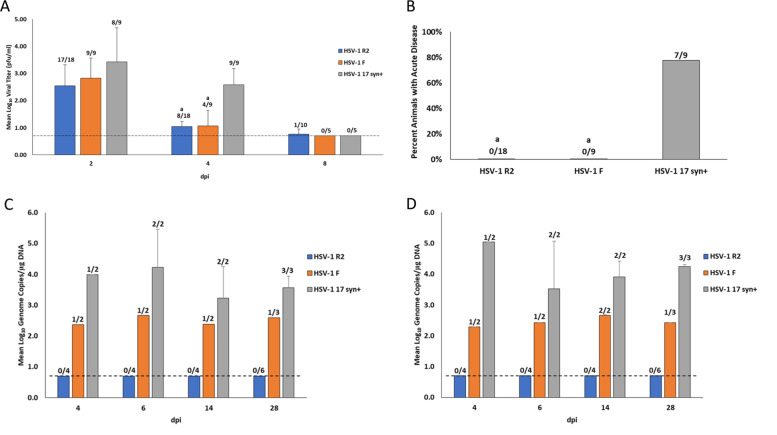
Pathogenesis of virus. Animals were inoculated intravaginally with either R2, parent strain F or wild-type strain 17 syn+. **A** Quantity (Log_10_ geometric mean titer (GMT) of vaginally shed virus for each day post inoculation (dpi), the number of animals with the detectable virus is shown above each bar. The dotted line indicates the limits of detection. Error bars are standard deviation. **B** Number of animals that developed the genital disease. **C** Log_10_ GMT of virus detected in the DRG. The number of animals with detectable virus in the DRG by qPCR on each day is shown above the bars. The dotted line indicates limits of detection. Error bars are standard deviation. **D** Log_10_ GMT of virus detected in the Spinal cord. a *P* < 0.005 vs 17syn+.

Following IVag inoculation, despite replicating with similar but slightly reduced kinetics in the vagina, (Fig. 1A) none of the animals inoculated with R2 or the parent F strain developed disease compared to 7 of 9 animals inoculated with HSV-1 strain 17 syn+ (Fig. 1B). Evidence of DRG infection, as evaluated by qPCR, revealed consistent infection by strain 17 syn+, about 50% infection in F strain inoculated animals but no detection of viral DNA in the DRG of any R2 inoculated animals (Fig. 1C). Almost identical findings were made in the spinal cords including no detection of the virus in any R2 inoculated animal (Fig. 1D). When combined 9 of 18 total neural specimens from animals infected with F strain had the detectable virus while none of the 36 samples from R2 infected animals had detectable virus in the DRG (*P* < 0.0001). When examined by explant co-cultivation, no virus was detected in any R2 or F infected animals but the virus was recovered in the DRG and spinal cord of 1 of 2, 17syn+ animals on day 6 and 14. Follow up studies of the remaining animals revealed recurrent disease in two of three infected with 17syn+ but none infected with F and R2 (*n* = 6).

### R2 vaccine efficacy

**Fig. 2:**
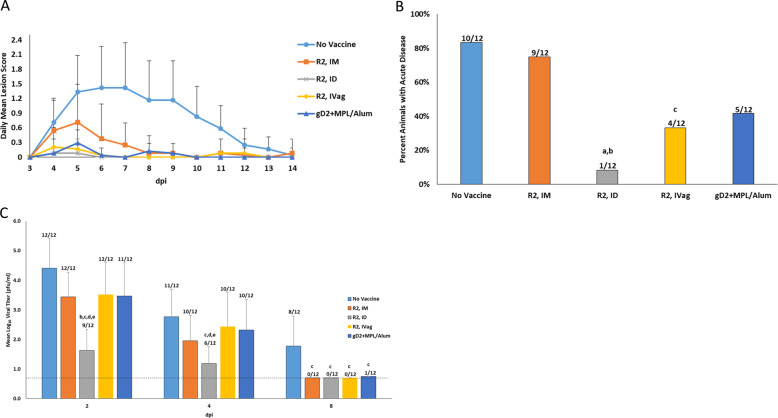
Effect of R2 vaccination by intramuscular (IM), intradermal (ID), and intravaginal (IVag) routes on acute disease following vaginal HSV-2 challenge. **A** The severity of acute disease (0–14 dpi) following intravaginal challenge with 1 × 10^6^ pfu of HSV-2 (MS Strain). IM vaccination with gD+MPL/Alum is included as a positive control. The severity of the acute disease was quantified using a score-scale ranging from 0 to 4. Error bars are standard deviation. **B** The percent of animals that developed acute genital lesions. The number of animals is indicated above each bar. **C** Replication of HSV-2 in the vaginal tract during the week after vaginal challenge. Animals were swabbed on days 2, 4, and 8 after challenge and shedding quantified by plaque titration. The numbers above the columns indicate the number of animals with the detectable virus. Bars are the Log_10_ GMT. Error bars are standard deviation. The dotted line indicates the level of detection. a *P* < 0.001 vs. No Vaccine, b *P* < 0.05 vs.R2 IM, c *P* < 0.05 vs. No Vaccine, d *P* < 0.05 vs R2 IVag *e* *P* < 0.05 vs. gD + MPL/Alum.

Guinea pigs were vaccinated with R2 by three routes and vaccine efficacy was analyzed following vaginal HSV-2 challenge. As shown in Fig. 2A, the R2 vaccine delivered by all routes and the gD2+MPL/Alum vaccinated group had significantly lower acute disease scores (*p* < 0.001) when compared to the No Vaccine group. The most effective route of R2 vaccination was ID, where the number of animals with any disease was significantly less compared to animals immunized with gD2+MPL/Alum (Fig. 2B). Similarly, animals vaccinated with R2 by the ID route had the largest decrease in vaginal virus replication: 99.9% reduction in peak titer (day 2) (Fig. 2C).

**Fig. 3:**
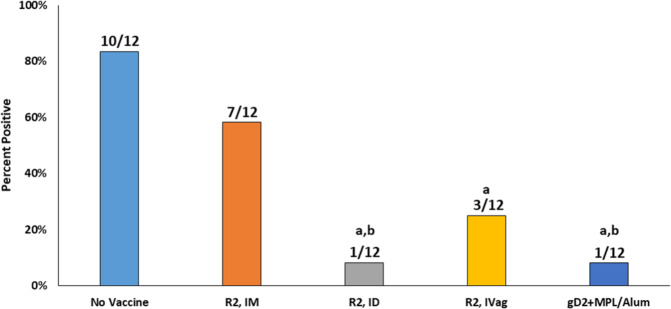
Effect of vaccination on the number of animals with latent HSV-2 challenge virus in the DRG at 63 dpi. Following the evaluation of animals for recurrent disease, animals were sacrificed, the DRGs obtained and the quantity of challenge virus was quantified by PCR. The number above the bar indicates the number of animals positive for challenge virus/total number of animals in each group. a *P* < 0.02 vs No Vaccine, b *P* < 0.03 vs R2 IM.

The effects of vaccination on the reduction of latent virus infection of the DRG is also an important goal of a prophylactic vaccine as it relates to recurrent disease and viral shedding. Latent HSV-2 was detected in the DRG in 10/12 animals in the No Vaccine group at 63 dpi (Fig. 3). The groups vaccinated with R2 by ID and IVag had a reduction in the number of animals with detectable latent HSV-2 virus (1/12 with ID and 3/12 with IVag) but the R2 IM vaccination group did not show a reduced number of latently infected animals (7/12). The reduction in DRG latent infection following vaccination with gD2 was the same as the R2 ID vaccinated group (1/12).

**Fig. 4:**
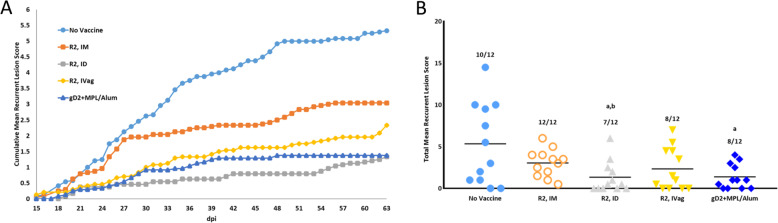
Effect of vaccination on the recurrent disease (days 15–63) following intravaginal challenge with HSV-2 MS strain. Animals were assessed daily for the presence of recurrent lesions. **A** Cumulative mean recurrent lesion score. **B** Total mean recurrent lesion score. The number above the bars is the number of animals with any recurrent disease while the bar shows the mean recurrent lesion score. Error bars are the S.D. a *P* < 0.01 vs No Vaccine mean recurrent lesion score, b *P* < 0.05 R2 IM animals with recurrent disease.

The reduction in neural infection was also reflected in a decrease in the number of animals with recurrent disease in the R2 ID animals and the mean recurrent lesion score (Fig. 4A, B). The mean recurrent lesion score was reduced from 5.3 ± 4.9 in the No Vaccine group to 1.3 ± 1.9 (*p* < 0.01) and 1.4 ± 1.5 (*p* < 0.01) for the R2 ID and gD2 + MPL/Alum groups, respectively (Fig. 4B). The IVag and IM routes were less effective.

**Fig. 5:**
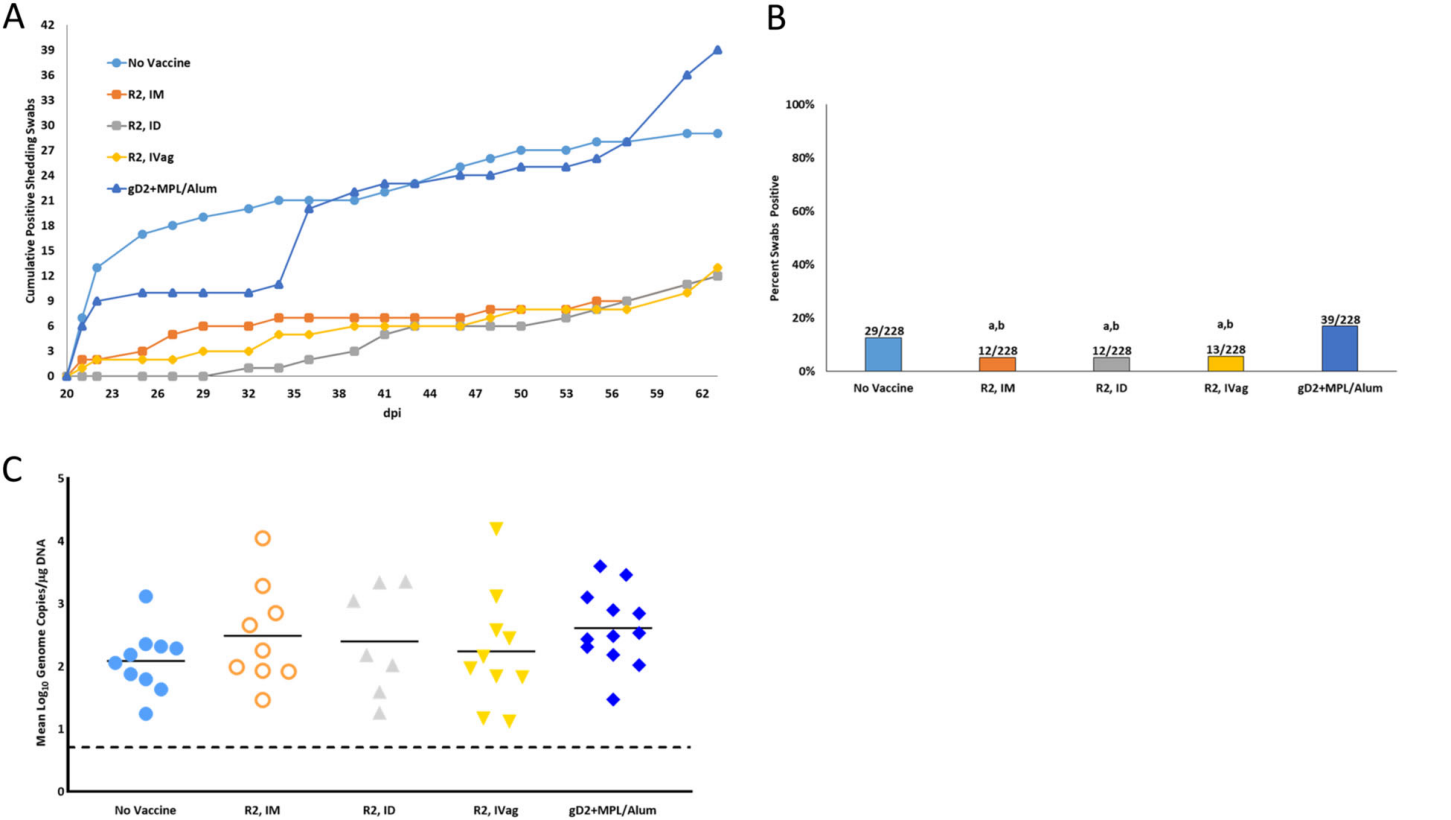
Effect of vaccination on recurrent virus shedding detected in vaginal swabs collected from day 21–63. Animals were swabbed three times a week for the presence of viral shedding. The number above is the number of positive days (swabs) / total number of swabs collected. **A** Cumulative shedding days. **B** Per cent positive days shed. The number at top of the bar is the number of positive swabs/total swabs. **C** Quantity (Log_10_ GMT) of the virus in swabs that were positive for the virus, Horizontal line is the mean. Each point represents an individual animal. a *P* < 0.01 vs No Vaccine, *b* P < 0.0002 vs gD2+MPL/Alum.

Importantly, R2 vaccination decreased the number of days with recurrent shedding compared to the No Vaccine group while gD2 + MPL/Alum had no effect on shedding days (Fig. 5A, B). R2 vaccination reduced virus shedding from 29 days during the monitoring period (12.7%) in the No Vaccine group to 12–13 days (5–6%). The decreased frequency of virus shedding appeared similar across all R2 groups and was statistically significant (Fig. 5A). The quantity of shed virus in the animals that shed appeared similar for all groups (Fig. 5C).

### Neutralizing antibody

**Fig. 6:**
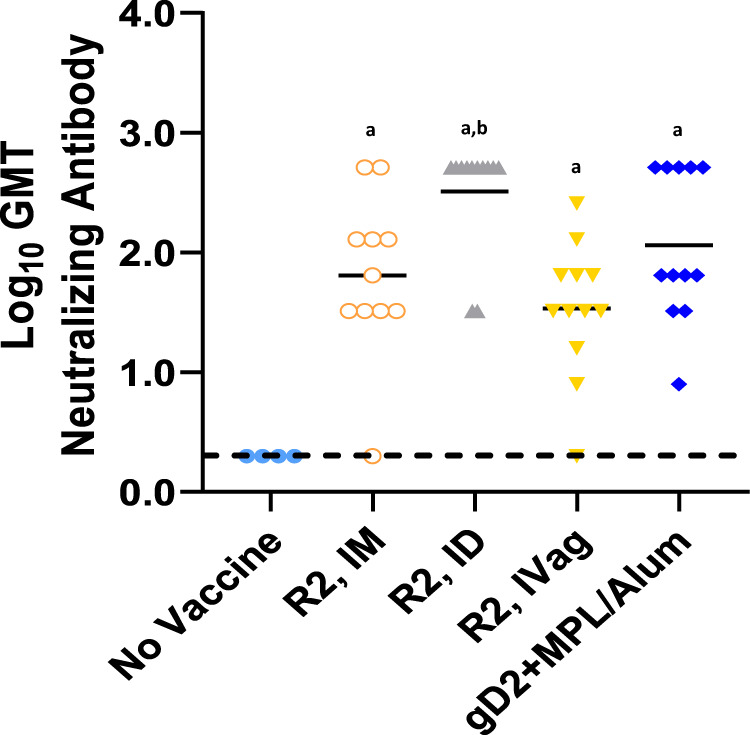
HSV-2 neutralizing antibody titers induced by three serial vaccinations. The sera dilution that showed a 50% or greater reduction in virus plaques compared to the virus/complement control was used and the Log_10_ geometric mean titer (GMT) is presented. Horizontal line is the mean. a *P* < 0.001 vs No Vaccine, b *P* < 0.03 vs R2 IM and R2 IVag.

Correlates of R2 protection, in particular neutralizing antibody, have not been previously examined. After three vaccinations, the neutralizing antibody titers, in the presence of complement, were significantly higher in the R2 groups compared to the No Vaccine group (*p* < 0.001) and were similar to the gD2 + MPL/Alum vaccinated group (Fig. 6). The highest titer was detected in the R2 ID vaccinated group (3-fold increase compared to gD2+MPL/Alum), corresponding to the increased effectiveness of this group.

## Discussion

The failure of the subunit gD2 vaccine in the recent clinical trial^14^ demonstrated that new approaches to HSV vaccines are needed, especially those that present multiple HSV antigens. R2 is an attractive candidate because it is specifically ablated for retrograde axonal transport and therefore fails to invade the peripheral and central nervous systems, as initially demonstrated in mice and validated here in guinea pigs^17^. The potential utility of a non-neuroinvasive HSV variant as a vaccine is highly regarded. However, early mutant viruses that were attenuated for neurotropism generally had broader replication defects in vivo that limited antigen presentation^19^. More recently, live-attenuated mutant viruses have been designed that reduce entry into neurons and show encouraging results as vaccine candidates^20–23^. The R2 design offers another solution to the challenge of producing a virus that replicates in the periphery inducing a strong immune response without seeding the nervous system and establishing a latent infection. Instead of adjusting tissue tropism, R2 selectively eliminates retrograde axonal transport, which is fundamental to neuroinvasion^17^. The design leaves all viral antigens intact for enhanced vaccine efficacy^17,24,25^.

Thus, selective elimination of retrograde delivery to the nervous system is an attractive approach for the development of HSV vaccines^26,27^ because it decreases concerns that a live-attenuated HSV vaccine could become latent, possibly revert or recombine, or subsequently cause complications if the immune system becomes compromised or distressed^3,26^. Similarly, the concerns that a persistent HSV vaccine virus could contribute to neurodegenerative diseases, such as Alzheimer’s disease, are eliminated^9,10^.

The R2 vaccine encodes a mutated UL37 gene^17^ The pUL37 protein contains three evolutionarily conserved surface-exposed regions: R1, R2, and R3^28^. Mutation of R2 produces viruses that propagate normally at peripheral sites of inoculation but fail to invade the nervous system by retrograde axonal transport, cannot establish a latent infection and are avirulent^17^. The ability to selectively eliminate the neuroinvasive property of these viruses, without otherwise impairing their replication and thus the induction of immunity, provides an intriguing immunization paradigm. The initial use of an HSV-1 R2 vaccine reflects the importance of HSV-1 in genital HSV infections and the importance of cross protection, as both HSV-1 and HSV-2 are important human pathogens. Evidence for cross protection can be found in many animal experiments. In the recent clinical trial, a gD2 vaccine was more protective against HSV-1 than HSV-2^14^. Nevertheless, an R2 derivative of HSV-2 will be produced to determine if vaccine efficacy is further enhanced.

In the experiments reported here, we first evaluated the attenuation of the R2 vaccine following vaginal inoculation of guinea pigs, reasoning that this is a sensitive and well-described model of HSV pathogenesis. Interestingly, although the parent F strain was attenuated relative to HSV-1 strain 17syn+ in guinea pigs as we have noted previously^26^, the R2 derivative was further attenuated, non-neuroinvasive, and avirulent. Thus, R2 was also not detected in the DRG either early during infection or at day 28, times when the parent F strain and wild-type strain 17 syn+ were readily detected.

We next evaluated the protection afforded by the R2 vaccine against vaginal HSV-2 challenge. R2 was administered by three routes (IM, ID, and IVag) and its efficacy was compared to a gD2+MPL/Alum vaccine, similar to the one used in clinical trials^14^. This comparison was intended to determine if R2 was at least as effective as the subunit vaccine and if R2 was more effective in the important outcomes regarding recurrent disease and recurrent virus shedding. Therefore, we used the guinea pig genital model because it uniquely allows for the evaluation of these critical endpoints. The advantages of this model are more fully discussed in a recent review^29^.

Protection was detected by R2 immunization using all 3 routes of vaccination but the ID route was consistently the most effective. Importantly, ID administration of the R2 vaccine-induced higher levels of neutralizing antibodies and provided greater protection than the gD2 subunit vaccine by several parameters. Perhaps, this was due to the improved neutralizing antiboy response and/or the ability of live-attenuated virus vaccines to induce T cell-mediated immunity that exceeds that of subunit vaccines. Given the encouraging findings with R2, a more detailed analysis of the correlates of protection is warranted and will be examined as part of future studies.

Evaluation of the acute disease revealed that only 1/12 of animals vaccinated with R2 by the ID route developed acute lesions compared to 10/12 with mock vaccinated animals. This compared favorably to 5/12 animals with the gD2 vaccine. IVag R2 vaccination was also effective (4/12), but to a lesser degree than the ID route. The increased protection following ID vaccination was also seen comparing acute vaginal replication, where the log_10_ geometric mean titer (GMT) of the virus was lowest in the ID group early after infection (*P* < 0.05 vs the gD2 vaccine). Protection of the DRG, the site of virus latency, was also detected along with a decrease in recurrent disease and recurrent virus shedding in some groups. For example, latent HSV-2 challenge virus was detected in only 1/12 animals immunized with R2 by the ID route or with gD2+MPL/Alum. Similarly, recurrent lesion scores were reduced from 5.3 to 1.3 and 1.4 in the R2 ID and gD2 groups, respectively.

Perhaps of greatest significance, recurrent virus shedding was reduced by 33–64% in each of the R2 vaccinated groups whereas the gD2+MPL/Alum group showed no benefit. This is of particular importance because recurrent virus shedding is the main means of HSV transmission. It should be noted that the qPCR assay used here detects HSV DNA, which is not necessarily an indication of infectious virus^30^. For example, in a previous experiment where 5–11% of vaginal guinea pig swabs were positive for HSV DNA none were culture positive^31^. Thus, the results obtained with R2 vaccination may be an underestimate of its potential to restrict shedding of the transmissible virus.

Because no HSV vaccines have yet proven effective clinically, it is not clear what endpoints of the guinea pig vaginal model will predict protection in human evaluations. In the experiments reported here R2 vaccination by the ID route protected the most animals from acute disease and had the largest decrease in acute virus replication. R2 vaccination also decreased latent virus detection in the DRG as well as recurrent disease and recurrent shedding. In comparison the gD2 MPL/alum vaccine did not significantly decrease the number of animals with acute disease, acute vaginal virus titers, or recurrent vaginal virus shedding. Only further clinical evaluations of vaccines that have been evaluated in this guinea pig model will allow a better understanding of predictability.

There are several anomalies that should be pointed out in our data. The most important is the discrepancy between the number of animals with latent virus detected in the DRG and recurrent shedding. This is most obvious in the gD2 MPL/alum group where vaccination markedly decreased the number of animals with the detectable latent virus and the recurrent lesion score but had no effect on the number of recurrent days with shed virus. It should be noted that there was a highly significant correlation between latent virus load and the recurrent lesion scores (*P* < 0.001) but not recurrent shedding days (*P* = 0.12) when all animals are included in the analysis. Further, similar findings were noted in the evaluation of another live-attenuated virus, VC2^26^, where VC2 reduced the number of animals with detectable virus in the DRG and recurrent shedding days while the gD2 MPL/Alum vaccine only reduced the number of animals with detectable virus in the DRG but not shedding days.

While we cannot currently reconcile these findings, one possible explanation is that gD2 MPL/Alum failed to protect against invasion and reactivation of HSV-2 from another latent site, perhaps autonomic neurons^32^. It is also possible that the immune response induced by sub-unit vaccines convert what would have been recurrent lesions into asymptomatic shedding episodes. Thus, the gD2 immunized group had fewer recurrent lesions but more recurrent shedding episodes. In any case, this could be an important limitation for sub-unit vaccines. The failure of the IM route to provide protection is also curious, as IM vaccination provided protection using another live-attenuated HSV-2 vaccine, VC2^26^. However, in the experiments provided here, the ID route induced significantly higher levels of neutralizing antibody compared to IM vaccination. It should also be noted that the ID route using gD2 MPL/Alum was not evaluated here and might provide improved protection compared to IM.

Although the use of ID vaccination is more often associated with killed vaccines such as influenza, evaluation of ID administration of modified vaccinia Ankara vaccine, a poxvirus vaccine, revealed a strong early local and systemic inflammatory response^33^. The ID route is also a potent inducer of tissue-resident T cells^34^ which may be important in protection from HSV infections^35,36^.

In summary, the live R2 HSV-1 vaccine is significantly attenuated in the guinea pig vaginal model and most importantly does not infect the nervous system within the limits of detection. R2 vaccination, especially by the ID route, provided protection against acute disease, recurrent disease and recurrent virus shedding that was equal or superior to the gD2+MPL/Alum vaccine. We consider these results highly encouraging, especially considering R2 is an HSV-1 virus and was evaluated for cross protection against HSV-2 challenge.

## Materials and methods

### Vaccines

The R2 recombinant virus, HSV1-GS6264, encodes five mis-sense mutations in the UL37 gene and was derived from a variant of the HSV-1 strain F infectious clone, pGS5923, as previously described^17,24^. HSV1-GS6264 also carries a single loxP site in the intergenic region between the UL3 and UL4 genes, but otherwise has all genes intact and does not encode other foreign sequences^25^.

The gD2 vaccine was prepared by G. Cohen (University of Pennsylvania) from Sf9 (Spodoptera frugiperda) cells (GIBCO BRL) infected with a recombinant baculovirus expressing gD2 as previously described^37,38^.

### Adjuvants

The MPL/Alum combination contained 50 μg of MPL (Sigma-Aldrich Corporation, St. Louis, MO) and 200 μg of Alhydrogel (2%) (Accurate Chemical and Scientific Corporation, Westbury, NY)^38^.

### Viruses and cells

HSV-2 strain MS (ATCC-VR540) was grown in low passage primary rabbit kidney cells and titered on rabbit kidney cell monolayers as previously described^39^. HSV-1 strains 17syn+ and F, and the R2 derivative of the latter, were grown in Vero cells^39^.

### Animals

Female Hartley guinea pigs (251–350 g) were obtained from Charles River Breeding Laboratories (Wilmington, MA) and housed under AAALAC approved conditions at Cincinnati Children’s Hospital Medical Center. All procedures and protocols were approved by the Cincinnati Children’s Hospital Research Foundation Animal Care and Use Committee.

### Study design and methods

The pathogenesis of R2 (*n* = 18), the parent F strain (*n* = 9), and the virulent HSV-1 strain 17 syn+ (*n* = 9) were initially determined by vaginally inoculating guinea pigs with 6.7 × 10^6^ PFU of each virus using the procedure described below. Primary disease was evaluated daily and vaginal virus replication on days 2, 4, and 8. Infection of the dorsal root ganglia (DRG) and spinal cord was evaluated for HSV-1 by qPCR and explant co-cultivation after sacrificing 2–6 animals/group on days 4, 6, 14, and 28, also as described below.

To evaluate protection from HSV-2 challenge, 60 Hartley guinea pigs (250–300 g) were divided into 5 groups (*n* = 12): group 1: no vaccine control, group 2: 1 × 10^6^ pfu R2 intramuscular (IM) into the quadriceps muscle, group 3: 1 × 10^6^ pfu R2 intradermal (ID) into the skin on the lower back, group4: 1 × 10^6^ pfu R2 IVag and group 5:5 μg gD2 + MPL/Alum IM. Animals were vaccinated 63, 42, and 21 days before the challenge. Animals were then challenged with HSV-2 MS strain intravaginally by using a pre-moistened calcium alginate swab (Puritan Calgiswab Type 3 Guilford, ME) to rupture the vaginal closure membrane of each animal. Using a 1 ml slip tip syringe, 1 × 10^6^ pfu of HSV-2 (MS strain) was then instilled into the vaginal vault in a 0.1 ml suspension^22^.

To determine acute vaginal virus replication, vaginal swabs were collected on select days and titers determined on Vero cells. During primary infection, a lesion score-scale was used ranging from 0 representing no disease to 4 representing severe vesiculoulcerative skin disease of the perineum^40^. The animals were then evaluated daily from 15 to 63 days post infection (dpi) for evidence of spontaneous recurrent herpetic lesions. The recurrent lesions are scored daily and presented as a cumulative daily score of the means for each animal over time. To assess recurrent virus shedding, animals were vaginally swabbed three times a week from day 21–63. We, however, acknowledge that the selection of day 21 as the starting point for recurrent shedding is somewhat arbitrary given the dynamic nature of the change from acute virus infection to reactivation events in neural tissue. The swabs were stored frozen (−80 °C), DNA was then extracted followed by measurement of HSV-2 DNA by qPCR analysis to determine the frequency of viral shedding into the genital tract. At the end of the study, the guinea pigs were sacrificed, and the spines were harvested and frozen (−80 °C) followed by dissection to obtain DRG for analysis of the latent virus burden using HSV-2 specific primers as described below.

### qPCR of HSV-1 and HSV-2 DNA

Viral levels of HSV-1 DNA in DRGs harvested at 4, 6, 14, and 28 dpi were determined as previously described^41^. In brief, the DRGs were homogenized on ice in 0.5 ml of 2% FBS Basal Medium Eagle. DNA was extracted from tissue homogenate using the QIAamp DNA Mini Kit (Qiagen #51306) according to the manufacturer’s protocol. The eluted DNA was quantified using a Nanodrop 2000 spectrophotometer and equal amounts of DNA were used to perform quantitative PCR using an Applied Biosystems 7500 Fast Real-Time PCR System. To maximize sensitivity, extracted DNA was evaluated at concentrations ranging from 100 to 800 ng. DNA previously purified from HSV-1 (McKrae) strain was used as a positive control and DNA previously extracted from an uninfected animal was used as a negative control. HSV-1 specific primers were used to detect HSV-1 (F strain) DNA. The primers and probe detect the HSV-1 gD gene and were obtained from Sigma-Aldrich and the sequences were as follows:

Forward: 5′-ACG/TAC/CTG/CGG/CTC/GTG/AAG/A-3′; reverse: 5′-TCA/CCC/CCT/GCT/GGT/AG-3′; and probe: 5′-FAM-AGC/CAA/GGG/CTC/CTG/TAA/GTA/CGC/CCT-tamRA-3′.

To determine the amount of HSV-2 shed and viral levels of HSV-2 in DRGs, HSV-2 gG2 gene detection was performed by quantitative PCR. The gG2 primer and probe sequences were as follows:

Forward: 5′-CGG/AGA/CAT/TCG/AGT/ACC/AGA/TC-3′; reverse: 5′-GCC/CAC/CTC/TAC/CCA/CAA/CA-3′; and probe: 5′-FAM-ACC/CAC/GTG/CAG/CTC/GCC/G-tamRA-3′.

Each PCR reaction contained 100 ng of DNA, 0.5 μM of each primer, 0.10 μM of FAM/tamRA fluorescent probe, and 10 μl of Taqman Gene Expression Master Mix (ABI) in a total volume of 20 μl reaction. PCR amplification of both HSV-1 and HSV-2 DNA was performed on a 7500 Fast Real-Time PCR system (ABI) using the following conditions: pre-incubation at 50 °C for 2 min and 95 °C for 10 mins followed by 40 cycles consisting of a denaturation step at 95 °C for 15 s, annealing at 60 °C for 1 min, and elongation at 72 °C for 10 s. A standard curve for each virus was generated with ten-fold serial dilutions of purified HSV-1 or HSV-2 DNA (ATCC) containing 10^5^–10^0^ HSV-2 copies in 50 ng of uninfected guinea pig brain DNA. The limit of detection for both HSV-1 and HSV-2 was determined to be 5 genome copies, with excellent linearity (*R* ≥ 0.98) over 5 logs of HSV genomic DNA content.

In order to confirm that the samples contained amplifiable DNA we amplified the housekeeping gene, GADPH from 10 animals with virus detected in the DRG and from 18 with no virus detected. All of the samples were positive for GADPH and had similar Ct values.

### Measurement of neutralizing antibody activity

In brief, sera samples obtained 21 days after the third vaccination were heat inactivated, serially diluted two-fold (1:4–1:2048) in media containing 10% rabbit complement (Cedarlane, Burlington, NC) and then mixed with HSV-2 (50–100 pfu) and incubated for 1 h at 37 °C^42^. The samples were then added to Vero monolayer and incubated at 37 °C for 1 h followed by a 1.5% methylcellulose overlay. After 3 days at 37 °C, the overlay was removed and plaques enumerated after staining with Crystal Violet (Sigma-Aldrich, St. Louis, MO). For each sample, the highest dilution producing a ≥50% reduction in plaques was considered the neutralizing antibody endpoint.

### Statistical analysis

For comparison of the means for two groups a Student’s *t* test was performed using two-tailed analysis. For comparison of multiple groups, an ANOVA was initially performed and if significant differences among all the groups was noted, a Tukey’s test to adjust for multiple comparisons was used. Data are presented as means and standard deviations. Incidence data were compared by Fishers’ exact test. A *P* value < 0.05 was considered significant.

### Reporting summary

Further information on research design is available in the Nature Research Reporting Summary linked to this article.

## Supplementary information

Reporting Summary

## Data Availability

The data sets generated and/or analyzed during the current study are available from the corresponding author.
