# Supplementary material for: The R2 non-neuroinvasive HSV-1 vaccine affords protection from genital HSV-2 infections in a guinea pig model
Source: NPJ Vaccines. 2020 Nov 6;5:104. doi: 10.1038/s41541-020-00254-8 (PMC7648054; doi:10.1038/s41541-020-00254-8)
Supplement: Supplementary file 1 — Reporting Summary [file 41541_2020_254_MOESM1_ESM.pdf]

nature research

Corresponding author(s): #00635R2
David Bernstein MD, MA

Reporting Summary

Nature Research wishes to improve the reproducibility of the work that we publish. This form provides structure for consistency and transparency in reporting. For further information on Nature Research policies, see Authors & Referees and the Editorial Policy Checklist.

Statistical parameters

When statistical analyses are reported, confirm that the following items are present in the relevant location (e.g. figure legend, table legend, main text, or Methods section).

n/a	Confirmed	
☐	☒	The exact sample size (n) for each experimental group/condition, given as a discrete number and unit of measurement
☐	☒	An indication of whether measurements were taken from distinct samples or whether the same sample was measured repeatedly
☐	☒	The statistical test(s) used AND whether they are one- or two-sided<br>*Only common tests should be described solely by name; describe more complex techniques in the Methods section.*
☒	☐	A description of all covariates tested
☐	☒	A description of any assumptions or corrections, such as tests of normality and adjustment for multiple comparisons
☐	☒	A full description of the statistics including central tendency (e.g. means) or other basic estimates (e.g. regression coefficient) AND variation (e.g. standard deviation) or associated estimates of uncertainty (e.g. confidence intervals)
☐	☒	For null hypothesis testing, the test statistic (e.g. F, t, r) with confidence intervals, effect sizes, degrees of freedom and P value noted<br>*Give P values as exact values whenever suitable.*
☒	☐	For Bayesian analysis, information on the choice of priors and Markov chain Monte Carlo settings
☒	☐	For hierarchical and complex designs, identification of the appropriate level for tests and full reporting of outcomes
☐	☒	Estimates of effect sizes (e.g. Cohen's d, Pearson's r), indicating how they were calculated
☐	☒	Clearly defined error bars<br>*State explicitly what error bars represent (e.g. SD, SE, CI)*

Our web collection on statistics for biologists may be useful.

Software and code

Policy information about availability of computer code

Data collection	No software was used
Data analysis	No software was used

For manuscripts utilizing custom algorithms or software that are central to the research but not yet described in published literature, software must be made available to editors/reviewers upon request. We strongly encourage code deposition in a community repository (e.g. GitHub). See the Nature Research guidelines for submitting code & software for further information.

Data

Policy information about availability of data

All manuscripts must include a data availability statement. This statement should provide the following information, where applicable:

- Accession codes, unique identifiers, or web links for publicly available datasets
- A list of figures that have associated raw data
- A description of any restrictions on data availability

Data Availability
The data sets generated and/or analyzed during the current study are available from the corresponding author.
Data Availability

The data sets generated and/or analyzed during the current study are available from the corresponding author.
Data Availability
The data sets generated and/or analyzed during the current study are available from the corresponding author.
Data Availability
The data sets generated and/or analyzed during the current study are available from the corresponding author.
Data Availability
The data sets generated and/or analyzed during the current study are available from the corresponding author.
Data Availability
The data sets generated and/or analyzed during the current study are available from the corresponding author.
The data sets Data Availability
The data sets generated and/or analyzed during the current study are available from the corresponding author.
The data sets generated and/or used during the current study are avaialble from the corresponding author.

Field-specific reporting

Please select the best fit for your research. If you are not sure, read the appropriate sections before making your selection.

☒ Life sciences ☐ Behavioural & social sciences ☐ Ecological, evolutionary & environmental sciences

For a reference copy of the document with all sections, see nature.com/authors/policies/ReportingSummary-flat.pdf

Life sciences study design

All studies must disclose on these points even when the disclosure is negative.

Sample size	Sample size was based on many years of experience with these models. Based on the variability of the endpoints a sample size of 12 animals/group has been found to be able to distinguish effective vaccine strategies from those not effective
Data exclusions	no exclusions
Replication	Reproducibility was confirmed by using a standard control vaccine and comparing results in this group to past experiments
Randomization	Anials are assigned to groups randomly
Blinding	Evaluators were not blinded as to group because of the limited number of evaluators available and the housing situation of the animals

Reporting for specific materials, systems and methods

Materials & experimental systems

n/a	Involved in the study	
☐	☒	Unique biological materials
☒	☐	Antibodies
☒	☐	Eukaryotic cell lines
☒	☐	Palaeontology
☐	☒	Animals and other organisms
☒	☐	Human research participants

Methods

n/a	Involved in the study	
☒	☐	ChIP-seq
☒	☐	Flow cytometry
☒	☐	MRI-based neuroimaging

Unique biological materials

Policy information about availability of materials

Obtaining unique materials	Vaccines are the property of Northwestern University

Animals and other organisms

Policy information about studies involving animals; ARRIVE guidelines recommended for reporting animal research

Laboratory animals	Harley strain guinea pigs

Wild animals	None
Field-collected samples	None
